# The long‐term effects of invasive earthworms on plant community composition and diversity in a hardwood forest in northern Minnesota

**DOI:** 10.1002/pei3.10075

**Published:** 2022-04-21

**Authors:** Genevieve Alexander, John Almendinger, Peter White

**Affiliations:** ^1^ Curriculum for the Environment and Ecology University of North Carolina – Chapel Hill Chapel Hill North Carolina USA; ^2^ Division of Forestry Minnesota Department of Natural Resources Grand Rapids Minnesota USA; ^3^ Department of Biology University of North Carolina – Chapel Hill Chapel Hill North Carolina USA

**Keywords:** hardwood forests, invasive earthworms, invasive species, *Lumbricus*, plant community composition

## Abstract

Nonnative European earthworms are invading hardwood forests of the Chippewa National Forest, MN. While effects on plant communities at the leading edge of invasion have been studied, little is known about longer‐term effects of invasive earthworms. We applied a model using historic O‐horizon soil thickness and a chronosequence approach to classify 41 hardwood sites in the Chippewa National Forest as “long‐term wormed” (wormed >2 decades), “short‐term wormed” or “unwormed/lightly wormed.” Graminoids, especially Carex pensylvanica, had the greatest mean percent cover in sites that had been wormed for over two decades. The families with the greatest negative change in mean percent cover after over two decades of earthworm invasion were Asteraceae, Violaceae, and Sapindaceae (specifically Acer species). Across all diversity metrics measured, long‐term wormed sites had the lowest understory plant species diversity, short‐term wormed sites had intermediate diversity, and unwormed/lightly wormed sites exhibited the highest diversity. Long‐term wormed sites had the lowest mean species richness across all sample scales (1–1024 m^2^). The greatest within‐group compositional dissimilarity occurred at sites that had been wormed for over two decades, suggesting that sites that had been wormed for over two decades have not reached a compositionally similar end‐state “wormed” community type. Our study suggests that understory diversity will decrease as hardwood forest stands become wormed over time. While our results support other findings that exotic earthworm invasion is associated with lower understory plant diversity in hardwood forests, our study was the first to use space‐for‐time substitution to document the effects after multiple decades of earthworm invasion.

## Graphical abstract

We applied a model using historic O‐horizon soil thickness and a chronosequence approach to classify 41 hardwood sites in the Chippewa National Forest as “long‐term wormed” (wormed >2 decades), “short‐term wormed” or “unwormed/lightly wormed”. Across all diversity metrics measured, long‐term wormed sites had the lowest understory plant species diversity, short‐term wormed sites had intermediate diversity, and unwormed/lightly wormed sites exhibited the highest diversity. The greatest within‐group compositional dissimilarity occurred at sites that had been wormed for over two decades, suggesting that sites that had been wormed for over two decades have not reached a compositionally similar end‐state “wormed” community type.

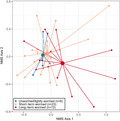

## 1 INTRODUCTION

European earthworms can have undesirable impacts on hardwood forests of the Great Lakes region in North America that were once devoid of earthworms due to glaciation (James, 1995; Resner et al., 2015). The establishment of invasive earthworms in these forests has been associated with a wide range of repercussions for hardwood forest ecosystems, including changes to ecosystem function (Dobson et al., 2017), ecosystem services (Frelich et al., 2019), and biodiversity (Ferlian et al., 2018). Recent studies suggest that invasive earthworms can affect hardwood forest plant communities both indirectly and directly. Indirectly, earthworms can alter soil structure and composition (Knowles et al., 2016), disrupt microbial networks (Dempsey et al., 2013), alter nutrient availability (Ferlian et al., 2020), and affect hydrological cycles (Catford, 2017). Directly, earthworms can ingest seeds (Cassin & Kotanen, 2016), consume roots and leaves (Curry & Schmidt, 2007), and modify seed bank composition (Nuzzo et al., 2015).

Although studies have assessed the initial effects of invasive earthworms on plant community composition across leading edges of earthworm invasion in hardwood forests of the Great Lakes region (Hale et al., 2006; Holdsworth et al., 2007), we are not aware of any studies that have used permanent plots to assess the effects of invasive earthworms on plant community composition in hardwood forests over multiple decades. However, one challenge of using permanent plots to study the long‐term effects of invasive earthworms is that study sites that have historic data on soils and plant communities generally lack information about historic earthworm presence. Here, we addressed this problem by creating a generalized linear model using the thickness of the O horizon to classify plots as wormed or unwormed when they were first sampled (Alexander et al., 2020). Although studies commonly use earthworm abundance or biomass to approximate earthworm impacts (Mahon & Crist, 2019; McCay & Scull, 2019), these two measures typically increase throughout the summer months. Because our sampling took place over multiple months, earthworm abundance and biomass were not appropriate for inclusion in the GLM model described in Alexander et al., 2020.

In this paper, we used permanent plots because it allowed us to classify plots by history of worm invasion. However, differences among observers meant that we could not make direct comparisons using historic vegetation data. Therefore, we used a space‐for‐time substitution approach to investigate differences among understory plant communities in plots that differed in the duration of worm invasion. To the best of our knowledge, our work is the first to analyze the effects of invasive earthworms on plant community composition and richness over multiple decades in the Great Lakes region. We sought to answer the following questions: (1) which plant species and families are the relative winners and losers as earthworms become established, and are there patterns in plant functional traits, such as mycorrhizal status or life form, that predict plant success? (2) What are the effects of invasive earthworms on understory plant diversity, and do these effects vary with scale? (3) What are the effects of invasive earthworms on plant community composition?

## 2 METHODS

### 2.1 Study area

**FIGURE 1:**
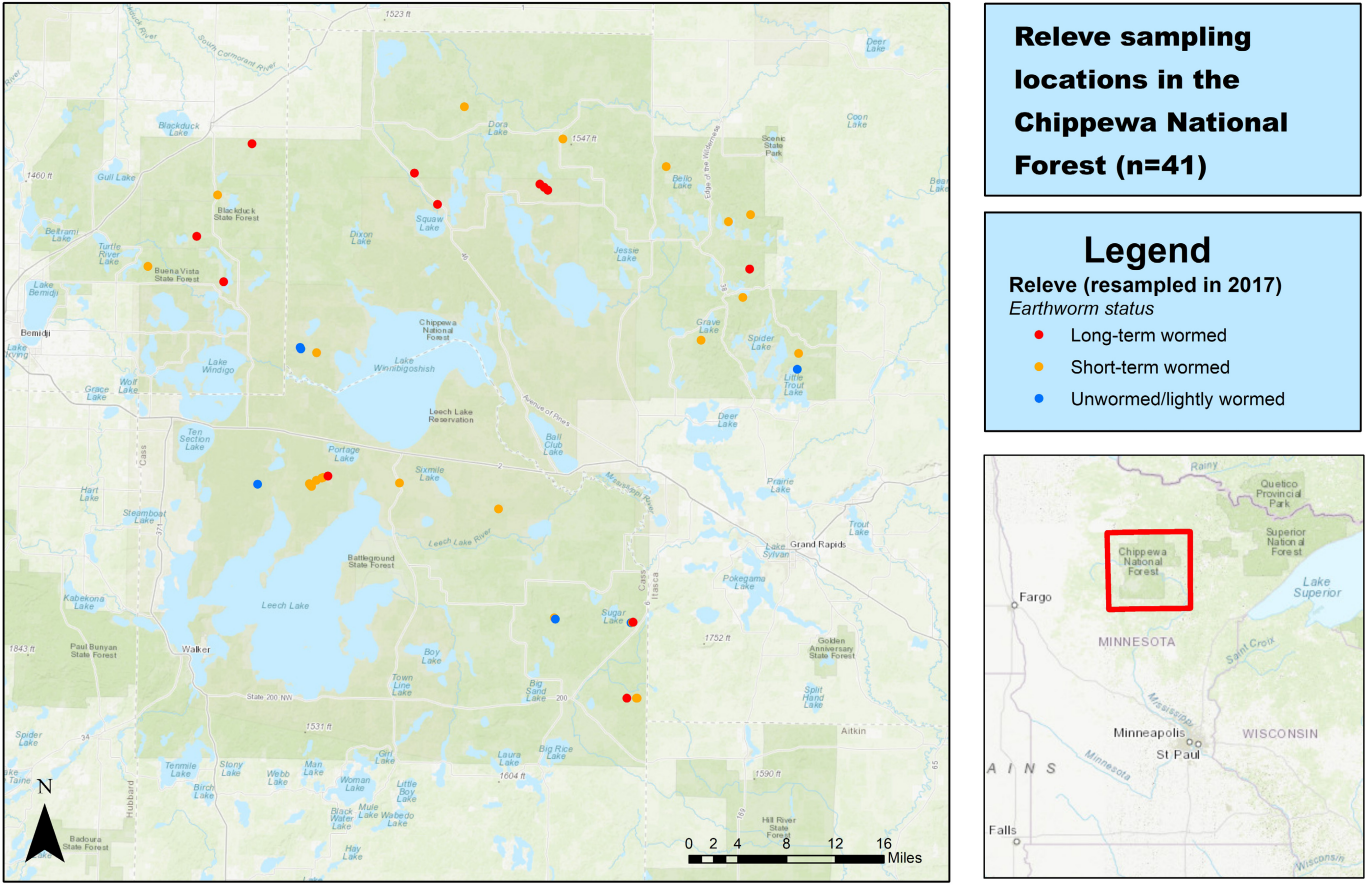
Map of relevé resampling locations in the Chippewa National Forest. Colored points represent 2017 relevé resampling locations (*n* = 41)

The Chippewa National Forest, located in north‐central Minnesota, contains permanent vegetation plots that were initially established and inventoried between 1989 and 1996 (Figure 1). Although these plots have historic data on soils and plant communities, no earthworm data were collected when they were initially sampled. In recent decades, nonnative European earthworms have been invading hardwood forest soils in the Chippewa National Forest through their widespread use as fishing bait and through transportation on logging roads and via horticultural soils (Hendrix & Bohlen, 2002).

Sampling took place within the three counties encompassing the forest: Cass, Beltrami, and Itasca. The Chippewa National Forest has a humid continental climate, with warm summers and cold winters. The mean monthly minimum temperature in the nearby town of Cass Lake is −20.8°C in January and the mean monthly maximum temperature is 19.3°C in July (Arguez et al., 2010). The mean monthly precipitation in Cass Lake is 2.2 inches (Arguez et al., 2010). The permanent plots selected for this study were limited to Mesic hardwood forests. Soils in this area are primarily well‐drained, loamy haplic glossudalfs (Richardson, 1997).

Historic permanent vegetation plots (relevés) were first established and sampled from early June to early September from 1989 to 1996 as part of a national effort within the U.S. Forest Service to map terrestrial ecosystems at several different scales (Cleland et al., 1997). On the Chippewa National Forest, this effort was completed in cooperation with the Minnesota County Biological Survey of the Minnesota Department of Natural Resources (Aaseng et al., 2011). In this study, a ‘relevé’ refers to a sampling unit and procedure modified from that first introduced by Braun‐Blanquet in the early 1900's (Braun‐Blanquet, 1932). During the initial 1989 to 1996 sampling period, representative samples of mature, homogeneous vegetation were targeted for plant community classification and vegetation characterization studies.

A subset of the relevés established in the Chippewa National Forest between 1989 and 1996 was inventoried between mid‐May and mid‐August 2017. In the selection process, we sought to reduce potential sources of variation other than worm impacts. We used four criteria: (1) we sampled only Northern Mesic Hardwood Forest or Northern Rich Mesic Hardwood Forest, (2) all plots had to have been unaffected by recent management (plots harvested between the two sampling periods were excluded and the age of re‐inventoried forest stands thus ranged from 50 to 137 years, with an average stand age of 90 years), (3) to distribute the plots throughout the study area, all plots were at a minimum of 10 chains (approx. 200 m) apart, and (4) surveyor, with priority given to relevés which were sampled by a surveyor with known soil science expertise. Within these criteria, we also sought out plots with a high likelihood of remaining unwormed because such sites have become increasingly rare. Thus, our study was not designed to assess the percent of relevés that were wormed or unwormed as an indicator of the overall rate of earthworm invasion or spread in the Chippewa National Forest. At one sampling location, a species‐area plot was not completed and therefore our analysis consisted of 41 relevés and 40 species‐area plots.

### 2.2 Sampling methodology

The modified relevé method as applied on the Chippewa National Forest involved establishing a 10 × 10‐m (100 m^2^) plot for subsequent soil and vegetation sampling. Relevés were re‐established by relocating magnets placed at the center of the relevé during the 1989 to 1996 sampling period using a metal detector. Methods for sampling soil horizons during the initial sampling period were replicated during the 2017 sampling season. Soil sampling involved digging a small soil pit (roughly 0.5 m^2^) and sampling soils to a depth of 30 cm. Thickness of the A‐horizon and О‐horizon were recorded in centimeters during both sampling periods, as was the texture and color of the A‐horizon. This soil sample was taken within a few meters outside of the permanent relevé in a random direction to avoid damaging vegetation inside the relevé. Inventorying plants in the 10 × 10‐m relevés involved recording all vascular plants and estimating their abundance using the Braun‐Blanquet cover/abundance scale (Table 1). Although the Minnesota Department of Natural Resources relevé protocol includes information about vegetation at all height strata, we limit our analyses to understory vegetation less than 2 m.

**TABLE 1:** Cover and abundance classifications for vegetation recorded in 10 × 10‐m relevés

Braun‐Blanquet cover class	Description	Cover Est. for analysis (geom. mean)
*r*	Single plant	1
+	2–20 plants	3
1	Many individuals	5
2	5–25%	11.2
3	25–50%	35.4
4	50–75%	61.2
5	75–100%	86.6

**FIGURE 2:**
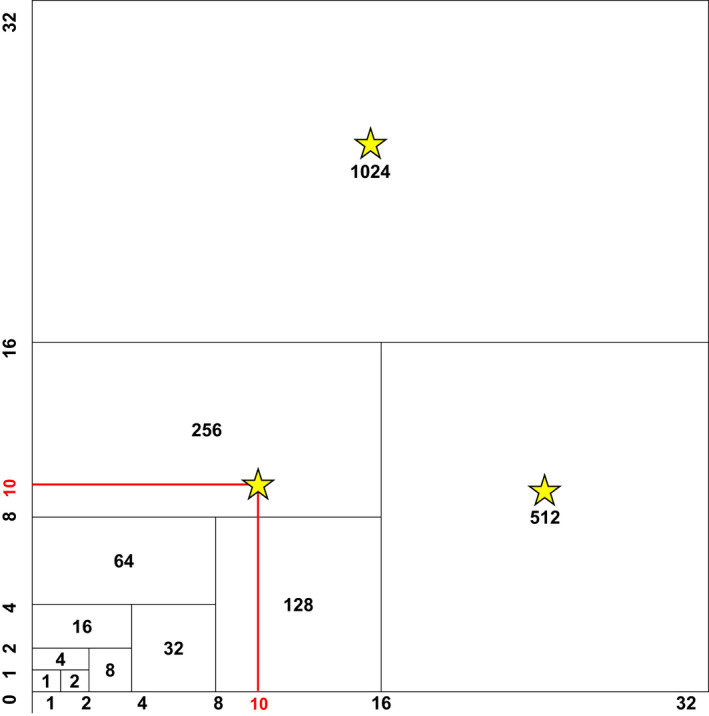
Sampling design for a 32 × 32‐m nested species‐area plot shown in black. Extent (10 × 10 m) of a relevé is shown in red. Yellow stars indicate approximate location of earthworm sampling locations

In addition to inventorying permanent 10 × 10‐m relevés, new 32 × 32‐m nested species‐area plots were established at permanent relevé locations in 2017 (*n* = 40, Figure 2). Species‐area plots were established as part of a statewide effort by the Minnesota Department of Natural Resources to understand species richness at multiple scales. The direction of these species‐area plots was expanded from the permanent 10 × 10‐m relevé was generally random, although we attempted to select a direction where the community type was uniform––for example, we chose not to have a species‐area plot cross a road or a wet depression. Starting at the 1 m^2^ nested plot and working outward to 1024 m^2^, we recorded the nested plot in which we first encountered each plant species. Thus, richness was recorded at each of 11 spatial scales (Figure 2).

Earthworms were sampled at three locations in each species‐area plot. The first earthworm sample was taken near the center of the 10 × 10‐m relevé in the 64‐m subplot (Figure 2). The second and third earthworm samples were taken near the middle of the 512 and 1024 m^2^ subplots. Earthworms were sampled in a 35 × 35‐cm square using the liquid mustard extraction method described in Hale (2013). Earthworms were anesthetized in isopropyl alcohol immediately in the field and then identified to species (or genus, when immature) and life stage within 10 h of collection. Abundance and biomass of earthworms were not calculated because earthworm abundance and biomass change throughout the summer sampling season. Because unwormed sites were considered high priority and generally sampled earlier in the season, estimates of earthworm abundance and biomass would not be appropriate to include in our analyses.

For data analysis purposes, a relevé or species‐area plot was considered ‘wormed’ if it was predominantly wormed (i.e., if 2/3 or 3/3 of earthworm samples in a site contained worms). A relevé or species‐area plot was considered “unwormed/lightly wormed” if it was predominantly unwormed (if 0/3 or 1/3 earthworm samples in a site contained worms). Of the unwormed/lightly wormed category, three sites had only epigeic earthworms (*Dendrobaena octaedra*) found in 1/3 earthworm samples, and three sites had no earthworms found in any earthworm samples. Relevés and species‐area plots with 1/3 samples containing worms likely represented a leading edge of earthworm invasion. Leading edges of earthworm invasion were classified as unwormed/lightly wormed (simply referred to as “unwormed” in Alexander et al., 2020) because earthworms had not yet become established and, as a result, plant communities and soil horizons may not yet have been strongly affected.

We created a generalized linear model as described in Alexander et al., 2020, which used thickness of the O‐horizon to predict whether Mesic hardwood sites in the Chippewa National Forest were wormed. The model was applied to data collected during the 1989 to 1996 sampling period to classify historic relevés as wormed or unwormed. Relevés that were wormed during the 1989 to 1996 sampling period and the 2017 sampling period are henceforth referred to as “long‐term wormed.” Relevés that were unwormed during the 1989 to 1996 sampling period and wormed in the 2017 sampling period are referred to as “short‐term wormed.” Relevés that were unwormed during the 1989 to 1996 sampling period and unwormed/lightly wormed during the 2017 sampling period are referred to as “unwormed/lightly wormed.” No sites were wormed during the 1989 to 1996 sampling period and unwormed during the 2017 sampling period.

In order to assess whether light levels in the understory affect understory vegetation, canopy cover was analyzed as an environmental variable. Canopy cover was estimated in the field as the average total cover of woody deciduous overstory vegetation from three points, estimated by the same three surveyors at each site. To determine whether canopy cover was significantly different among the three worm categories, we used a one‐way analysis of variance (ANOVA) test.

Prior to analyses, some taxa for which field identification to species was particularly challenging were grouped together at the generic (e.g.,: *Amelanchier*) or subgeneric (e.g.,: *Viola pubescens/canadensis*) level. All statistical analysis was conducted in R Studio (R Core Team, 2020). For all understory plant species or plant families that occurred in greater than five relevés, we created a table of mean percent cover for unwormed/lightly wormed, short‐term wormed, and long‐term wormed relevés. We used understory Küchler life form cover data from the 10 × 10‐m relevés to assess whether life forms (woody evergreen, woody deciduous, forbs, and graminoid plant species) explained understory differences among the three worm groupings.

### 2.3 Statistical analyses

Mean percent cover was used to assess differences in species, family, and Kuchler life forms among unwormed/lightly wormed, short‐term wormed, and long‐term wormed plant communities in 10 × 10‐m relevés. The Kruskal–Wallis H test was used to test the significance of mean percent cover differences among earthworm categories.

Four biodiversity indices (Shannon–Wiener, inverse Simpson's index, Pielou's evenness, and species richness) were used to assess differences in diversity among unwormed/lightly wormed, short‐term wormed, and long‐term wormed plant communities in 10 × 10‐m relevés. Shannon–Wiener diversity is more sensitive to both species richness and species evenness than Simpson's index. Because Simpson's index decreases as diversity increases, we reported the inverse Simpson's index (1/D). Species richness was calculated as the number of understory plant species per relevé, or the number of total species per species‐area plot. A one‐way ANOVA with pairwise comparisons was conducted to test the significance of mean Shannon–Wiener diversity, Simpson's index, and species richness among unwormed/lightly wormed, short‐term wormed, and long‐term wormed relevés.

We created a species accumulation curve for each of our three earthworm categories: unwormed/lightly wormed, short‐term wormed, and long‐term wormed species‐area plots. The curves describe the cumulative count of species per increase in area sampled for species‐area plot data. We used a logarithmic linear model to plot the line of best fit with the “ggplot2” package in R (Wickham, 2011).

Nonmetric multidimensional scaling (NMS) was used to ordinate plant species and sites and to explore gradients in plant species composition in relation to earthworm presence in the Chippewa National Forest. NMS is an ordination tool with several unique properties, including being the only ordination technique where sample separation is directly linked to sample dissimilarity. NMS does not assume linearity, it does not presume an underlying model of species response, and it does not assume an inherent dimensionality of the data. In NMS, the user chooses a set number of axes for ordination prior to analysis, and the data are subsequently fitted to these dimensions (Kruskal 1964). The NMS ordination was created using understory quantitative vegetation data collected in 2017. We created an NMS ordination using the 2017 10 × 10‐m relevé data with the “vegan” package in R (Oksanen et al., 2020). Prior to ordination, data were transformed using a Wisconsin double standardization and square root transformation. A Bray–Curtis dissimilarity matrix was used for NMS analysis. After selecting an appropriate dimensionality for the NMS analysis, a 30 iteration NMS ordination with random starts was run and the iteration with the lowest stress level was selected. The NMS configuration was rotated to orient axes that explained the most variance as the primary axes. Finally, sites were added as points on the NMS plot and categorized by worm grouping.

The “envfit” function in the “vegan” package was used to assess correlations between a subset of environmental variables, earthworm assemblages, and NMS axes. The environmental variables that were tested for correlation with NMS axes include Easting, Northing, elevation, slope, 1989–1996 A‐ horizon thickness, 1989–1996 O‐horizon thickness, 2017 A‐horizon thickness, 2017 O‐horizon thickness, and 2017 overstory cover. Easting and Northing were tested for correlation with NMS axes in to examine any geographic variation. Earthworm assemblages were tested for correlations with NMS axes because different earthworm assemblages can have different effects on soils and plants (Frelich et al., 2006). The 2017 earthworm‐related variables that were tested for correlation with NMS axes include earthworm presence, epigeic earthworm presence, epi‐endogeic earthworm presence, endogeic earthworm presence, anecic earthworm presence, epi‐endogeic/anecic earthworm presence (juvenile *Lumbricus* species), and sum of earthworm eco‐groups.

Compositional differences between unwormed/lightly wormed, short‐term wormed, and long‐term wormed plant communities were tested using the permutational multivariate analysis of variance method (perMANOVA) described in Anderson (2001). This was accomplished using the “adonis2” function of the “vegan” package in R (Oksanen et al. 2018). The perMANOVA was calculated using a Bray–Curtis dissimilarity matrix applied to the 2017 understory relevé and species‐area plant data and 100,000 permutations. A post hoc pairwise comparison of the perMANOVA results was conducted with a false discovery rate (FDR) correction and 100,000 permutations using the “pairwise. adonis” function in R (Martinez Arbizu, 2017).

## 3 RESULTS

Locations of relevés resampled in 2017 are depicted in Figure 1. Out of the 41 relevés originally established from 1989 to 1996 and resampled in 2017, six relevés were classified as unwormed/lightly wormed, 23 relevés were classified as short‐term wormed, and 12 relevés were classified as long‐term wormed. Earthworm species identified in summer 2017 sampling included *Lumbricus terrestris* (anecic), *Lumbricus rubellus* (epi‐endogeic), *Apporectodea* spp. (endogeic), *Octolasion* spp. (endogeic), *Dendrobaena octaedra* (epigeic), *Eiseniella tetraedra* (epigeic), and *Dendrodrilus rubidus* (epigeic). We performed a one‐way ANOVA test to ensure that observed differences in plant communities among unwormed/lightly wormed, short‐term wormed, and long‐term wormed relevés could not simply be ascribed to differences in canopy cover among the three worm categories. We found no statistically significant differences in canopy cover among the three worm categories [*F*(2, 38) = 1.07, *p* = 0.352].

### 3.1 Winners and losers

Woody deciduous species had similar mean percent cover for unwormed/lightly wormed, short‐term wormed, and long‐term wormed relevés (Appendix 1). Woody evergreen species had low mean percent cover at all relevés, with the greatest mean cover at unwormed/lightly wormed sites and the smallest mean percent cover at long‐term wormed sites. The largest differences in mean percent cover occurred with graminoid species, with over 30% lower mean percent cover of graminoids in unwormed/lightly wormed (11.7%) compared to long‐term wormed (45.0%) relevés. Forbs had the highest mean percent cover in short‐term wormed relevés, and the lowest mean percent cover in long‐term wormed relevés.

The families Cyperaceae and Sapindaceae occurred in all sampled relevés (Table 2). The greatest relative change in mean percent cover among families occurred for the Cyperaceae family, which had a mean cover of 7.4% in unwormed/lightly wormed relevés and a mean cover of 40.0% in long‐term wormed relevés.

**TABLE 2:** Percent cover of understory plants by family and worm category for unwormed/lightly wormed, short‐term wormed, and long‐term wormed relevés. The “spp” column represents the number of species (or species complex) per family

Percent cover of understory plants by family and worm category					
Family	Spp	*n*	Unwormed/Lightly wormed	Short‐term wormed	Long‐term wormed
Adoxaceae* (*p* = 0.024)	4	13	0.5	1.4	0.8
Apiaceae* (*p* = 0.003)	4	30	4.0	3.6	3.0
Araceae* (*p* = 0.006)	1	15	0.0	1.1	1.7
Araliaceae* (*p* < 0.001)	2	34	4.0	4.3	2.6
Aristolochiaceae* (*p* = 0.016)	1	12	0.5	1.8	0.7
Asteraceae* (*p* < 0.001)	13	35	6.7	5.0	3.8
Betulaceae* (*p* < 0.001)	5	40	7.5	8.2	6.8
Caprifoliaceae* (*p* < 0.003)	2	27	2.0	2.1	2.2
Colchicaceae* (*p* < 0.001)	3	36	7.2	10.7	6.6
Cornaceae* (*p* = 0.001)	3	31	3.0	2.6	2.6
Cyperaceae* (*p* < 0.001)	8	41	7.4	18.2	40.0
Dennstaedtiaceae (*p* = 0.116)	1	12	1.0	1.1	0.9
Dryopteridaceae* (*p* < 0.001)	5	30	3.0	6.7	2.5
Equisetaceae (*p* = 0.194)	4	9	0.5	0.5	2.1
Ericaceae (*p* = 0.494)	5	11	1.0	0.6	1.3
Fabaceae* (*p* = 0.038)	4	20	2.8	2.3	1.7
Fagaceae* (*p* = 0.005)	2	32	3.8	2.8	2.8
Grossulariaceae* (*p* = 0.001)	2	23	2.2	2.1	1.3
Liliaceae* (*p* < 0.001)	1	33	3.3	3.5	3.4
Lycopodiaceae* (*p* = 0.023)	4	13	0.5	1.7	1.2
Malvaceae* (*p* = 0.008)	1	28	2.8	2.4	1.8
Melanthiaceae (*p* = 0.678)	3	12	0.8	0.6	0.8
Myrsinaceae* (*p* < 0.001)	1	27	2.3	2.8	1.6
Oleaceae (*p* = 0.076)	2	24	1.7	1.4	2.0
Orchidaceae* (*p* < 0.001)	6	11	0.0	1.2	0.3
Ophioglossaceae* (*p* < 0.001)	1	22	1.5	2.0	0.7
Osmundaceae* (*p* = 0.003)	1	10	0.5	1.4	0.1
Pinaceae* (*p* = 0.001)	3	15	1.5	1.0	0.1
Poaceae* (*p* < 0.001)	11	35	3.8	4.8	4.9
Ranunculaceae* (*p* < 0.001)	4	39	8.2	7.1	6.9
Rosaceae* (*p* = 0.013)	7	31	6.0	4.8	6.3
Rubiaceae (*p* = 0.125)	2	18	1.5	1.3	1.2
Ruscaceae* (*p* < 0.001)	3	40	6.2	8.6	7.5
Salicaceae* (*p* = 0.005)	2	18	0.5	1.5	2.3
Sapindaceae* (*p* < 0.001)	3	41	16.4	16.5	14.7
Thymelaeaceae* (*p* < 0.001)	1	28	1.2	2.2	3.1
Violaceae* (*p* < 0.001)	3	29	3.8	3.2	2.0

*Note*: Sample size (*n*) represents the number of relevés that each family was found. Families with less than nine occurrences were excluded from the table. Data were collected from 41 10 × 10‐m relevés during summer 2017 in the Chippewa National Forest. Significance was tested using the Kruskal–Wallis H test (df = 2).

*Acer saccharum*and *Carex pensylvanica* occurred in all sampled relevés (Appendix 1). The greatest relative difference in mean percent cover among species occurred for *Carex pensylvanica*, which had a mean cover of 5% in unwormed/lightly wormed relevés and a mean cover of 35.8% in long‐term wormed relevés. In unwormed/lightly wormed relevés, the top four understory plants by mean percent cover in order of abundance were *Acer saccharum*, *Carex pensylvanica*, *Corylus cornuta*, and *Anemone quinquefolia*. Understory plants with the greatest mean percent cover in short‐term wormed relevés were *Carex pensylvanica*, followed by *Acer saccharum*, *Uvularia grandiflora*, and *Athyrium filix‐femina*. The top four understory plants by mean percent cover in long‐term wormed relevés were *Carex pensylvanica*, *Acer saccharum*, *Maianthemum canadense*, and *Oryzopsis asperifolia*. When comparing species with >5 occurrences, 34 understory plant species had greater mean percent cover at unwormed/lightly wormed relevés than long‐term wormed releves (Appendix 1). There were 27 understory plant species that had greater mean percent cover at long‐term wormed relevés than unwormed/lightly wormed relevés.

### 3.2 Diversity

**FIGURE 3:**
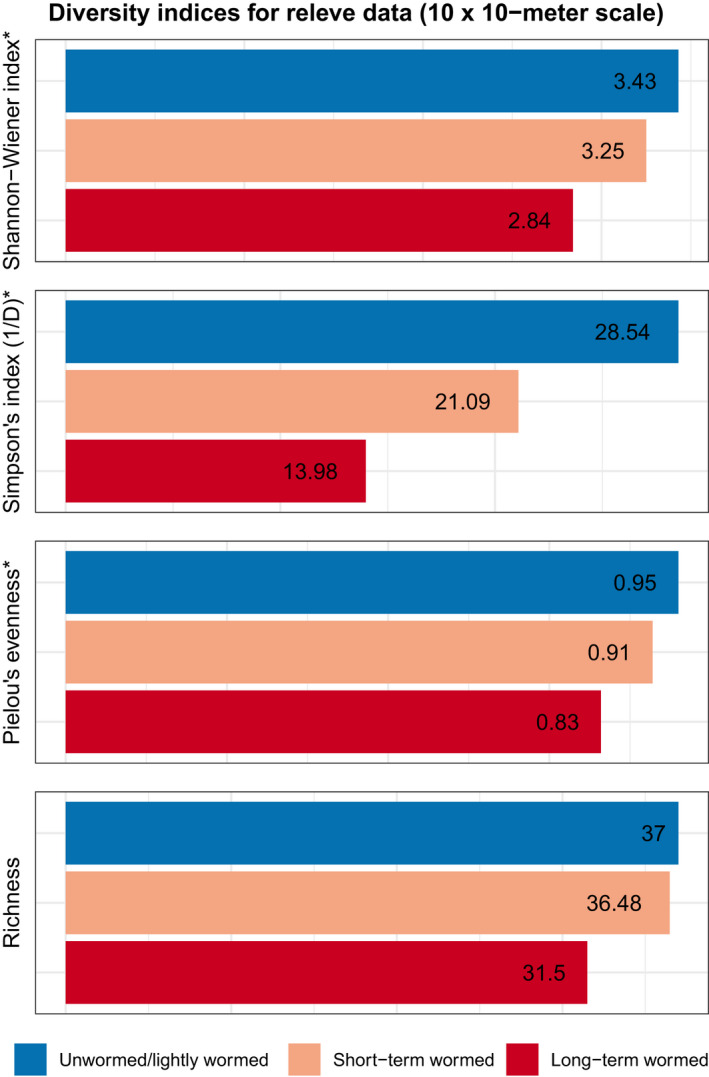
Diversity indices for unwormed/lightly wormed, short‐term wormed, and long‐term wormed plant communities from 10 × 10‐m relevé data collected in hardwood forests of the Chippewa National Forest in 2017. Indices with an asterisk (*) represent diversity values which were statistically significantly different among the three worm status categories

Across all measures of diversity (Shannon–Wiener, inverse Simpson, Pielou's evenness, and species richness), unwormed/lightly wormed sites had the highest diversity values and long‐term wormed sites experienced the lowest diversity values (Figure 3). A one‐way ANOVA test indicated statistically significant differences in Shannon–Wiener diversity among the three worm categories [*F*(2, 38) = 3.65, *p* = 0.036)]. A post hoc pairwise *t*‐test with an FDR (false discovery rate) p‐value correction revealed statistically significant differences between Shannon–Wiener diversity in unwormed/lightly wormed and long‐term wormed relevés (*p* = 0.044) and in short‐term wormed and long‐term wormed relevés (*p* = 0.04), but not in unwormed/lightly wormed and short‐term wormed relevés (*p* = 0.419). A one‐way ANOVA test indicated statistically significant differences in inverse Simpson's diversity among the three worm categories [*F*(2, 38) = 3.432, *p* = 0.042]. A post hoc pairwise *t*‐test with an FDR p‐value correction revealed a statistically significant difference between inverse Simpson's diversity in the unwormed/lightly wormed and long‐term wormed relevés (*p* = 0.045). Simpson's diversity was not statistically significantly different in unwormed/lightly wormed and short‐term wormed relevés (*p* = 0.164), or in short‐term wormed and long‐term wormed relevés (*p* = 0.134). Pielou's evenness was significantly different among worm categories [*F*(2, 38) = 4.450, *p* = 0.018]. A post hoc pairwise *t*‐test with an FDR p‐value correction revealed significant differences between Pielou's evenness in the unwormed/lightly wormed and long‐term wormed relevés (*p* = 0.025), and between short‐term wormed and long‐term wormed relevés (*p* = 0.025). Mean understory plant species richness was highest at unwormed/lightly wormed relevés (37), slightly lower at short‐term relevés (36.5), and lowest at long‐term wormed relevés (31.5). A one‐way ANOVA test did not indicate statistically significant differences in mean understory plant species richness among the three worm categories [*F*(2, 38) = 1.089, *p* = 0.347].

### 3.3 Species richness and scale

**FIGURE 4:**
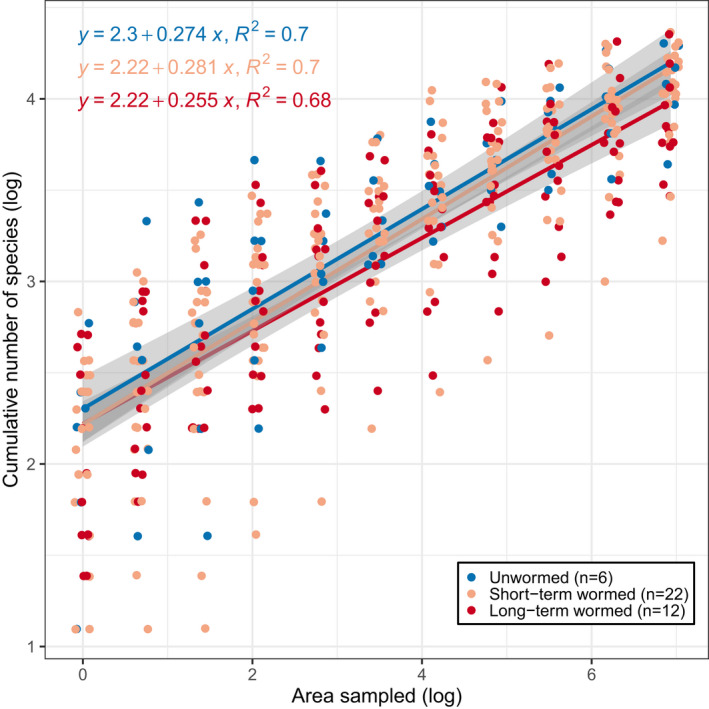
Species accumulation curve for unwormed/lightly wormed, short‐term wormed, and long‐term wormed plant communities from species‐area plot data collected in hardwood forests of the Chippewa National Forest in 2017. Gray fill indicates 95% confidence interval

Species accumulation curves indicated that long‐term wormed plots had the lowest mean richness at all measured scales (1–1024 m^2^, Figure 4). Short‐term wormed plots had similar low mean richness as long‐term wormed plots at the smallest scale, but at the largest scale measured (1024 m^2^), short‐term wormed plots had mean richness similar to unwormed/lightly wormed plots (Figure 4). At 1024 m^2^, unwormed/lightly wormed sites had the greatest mean total number of species (60), when compared to short‐term wormed (58), and long‐term wormed (50).

### 3.4 Plant community composition

**FIGURE 5:**
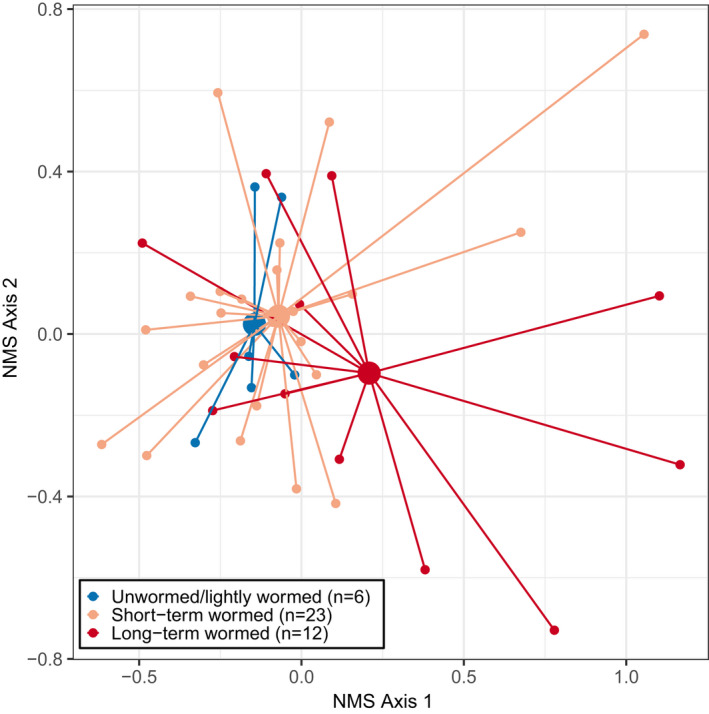
NMS plot for unwormed/lightly wormed, short‐term wormed, and long‐term wormed plant communities from 10 × 10‐m relevé data collected in hardwood forests of the Chippewa National Forest in 2017. Larger circles represent the centroid for each community type. Smaller circles represent individual species‐area plots. Stress for the NMS ordination was 0.17

An optimal NMS solution with two dimensions and a stress level of 0.17 was selected for the 2017 relevé understory vegetation data (Figure 5). Environmental variables that were significantly correlated with NMS axes include Easting, Northing, elevation, 1989–1996 A‐horizon thickness, and 2017 A‐horizon thickness (Table 3). Earthworm‐related variables that were significantly correlated with NMS axes include anecic earthworm presence, epi‐endogeic/anecic earthworm presence (juvenile *Lumbricus* species), and sum of earthworm eco‐groups. As sites became more wormed, they appeared to cluster toward the right side of the NMS ordination, which was significantly associated with Northing, 1989–1996 A‐horizon thickness, 2017 A‐horizon thickness, anecic earthworm presence, epi‐endogeic/anecic earthworm presence, and sum of earthworm eco‐groups.

**TABLE 3:** Correlations between environmental and earthworm variables and NMS ordination axes. Data collected from 10 × 10‐m relevés in Mesic hardwood forests of the Chippewa National Forest in summer 2017

Environmental variable	NMS1	NMS2	*r* ^2^	Pr(>*r*)
Easting	0.67	0.74	0.16	0.04*
Northing	0.97	−0.22	0.23	0.01 **
Elevation	0.20	0.98	0.19	0.02 *
Overstory cover (2017)	−0.04	1.00	0.14	0.06
Slope	−0.09	1.00	0.00	1.00
A‐horizon thickness (1989–1996)	0.88	−0.47	0.43	0.00 ***
O‐horizon thickness (1989–1996)	−0.93	0.36	0.06	0.31
A‐horizon thickness (2017)	0.60	−0.80	0.18	0.02 *
O‐horizon thickness (2017)	−0.65	0.76	0.14	0.06

Earthworm variable (2017)	NMS1	NMS2	*r* ^2^	Pr(>*r*)
Earthworm presence	0.96	−0.29	0.02	0.65
Epigeic earthworm presence	0.96	−0.29	0.02	0.65
Epi‐endogeic earthworm presence	0.93	−0.37	0.07	0.28
Endogeic earthworm presence	0.33	−0.95	0.03	0.57
Anecic earthworm presence	0.99	−0.11	0.27	0.00**
Epi‐endogeic/anecic earthworm presence	0.98	−0.22	0.19	0.02*
Sum of earthworm eco‐groups	0.93	−0.36	0.16	0.04*

*Note*: Signif. codes: 0 ‘***’ 0.001 ‘**’ 0.01 ‘*’ 0.05 ‘.’ 0.1 ‘’ 1; Number of permutations: 10,000.

Overall, within‐group compositional dissimilarity (0.565) was smaller than between‐group compositional dissimilarity (0.582). Unwormed/lightly wormed sites displayed the lowest within‐group (0.480) and overall dissimilarity (i.e., the greatest similarity). Mean dissimilarity between unwormed/lightly wormed and short‐term wormed sites was second lowest (0.523). Group dissimilarity was greatest between short‐term and long‐term wormed releves (0.609).

The significance of compositional differences among unwormed/lightly wormed, short‐term wormed, and long‐term wormed plant communities was tested using the permutational multivariate analysis of variance method (perMANOVA). Compositional differences among the three worm categories were significant [100,000 permutations, *F*(2, 38) = 2.154, *p* = 0.006]. A post hoc pairwise comparison was conducted to determine individual differences among unwormed/lightly wormed, short‐term wormed, and long‐term wormed relevés. Pairwise perMANOVA tests with an FDR correction and 100,000 permutations revealed significant compositional differences between short‐term and long‐term wormed sites (Table 4), as well as between unwormed/lightly wormed and long‐term wormed sites (Table 4). Compositional differences between unwormed/lightly wormed and short‐term wormed sites were not statistically significant (Table 4).

**TABLE 4:** Results of pairwise comparisons (PERMANOVA) for unwormed/lightly wormed, short‐term wormed, and long‐term wormed plant communities from 10 × 10‐m relevé data collected in hardwood forests of the Chippewa National Forest in 2017

Pairwise perMANOVA grouping	F model	*R* ^2^	*p*‐value	*P*adj sig
Short‐term wormed vs. unwormed/lightly wormed	0.782	0.028	0.723	0.723
Short‐term wormed vs. long‐term wormed	3.024	0.084	**0.003**	**0.009**
Unwormed/lightly wormed vs. long‐term wormed	2.550	0.147	**0.028**	**0.043**

*Note*: Results are calculated with a false discovery rate (FDR) correction and 100,000 permutations. Significant results are highlighted in bold.

## 4 DISCUSSION

Taken together, these results indicate that earthworm invasion is associated with reduced species diversity and altered composition in forest understories and, further, that worming effects increase with the duration of earthworm invasion such that even sites wormed for more than two decades are still experiencing species loss and composition shifts.

### 4.1 Winners and losers

Differences in abundance of understory plant species after earthworm invasion followed similar distributional patterns as was reported by Hale et al. (2006). Of the relevés sampled in 2017, *Carex pensylvanica* had a sevenfold difference in cover among the wormed categories. Unlike the majority of native understory plants that are present in sugar maple––basswood forests of the Great Lakes region, *Carex pensylvanica* is non‐mycorrhizal (Brundrett & Kendrick, 1988). Non‐mycorrhizal plant species may receive a relative competitive advantage after earthworms invade and disrupt existing mycorrhizal networks.

*Arisaema triphyllum*is a native forb that possesses calcium oxalate crystals in its foliage and roots. These crystals may deter above and belowground herbivores, including earthworms. Hale et al. (2006) observed that *Arisaema triphyllum* became a dominant understory plant species in heavily wormed sites with a high biomass of *Lumbricus rubellus* earthworms, however they hypothesized that *Arisaema triphyllum* would experience declines in population as earthworms became established over time. We did not observe a relative dominance of *Arisaema triphyllum* in hardwood forest relevés that became wormed, although the mean percent cover of this species was indeed lowest in unwormed/lightly wormed relevés (no occurrences). Unlike the hypothesized decrease in *Arisaema triphyllum* abundance over time suggested by Hale et al. (2006), our results indicate that *Arisaema triphyllum* continues to experience a competitive advantage in hardwood forests that have been wormed for multiple decades, reaching its highest mean percent cover in long‐term wormed sites.

The understory plant species associated with the greatest negative change in mean percent cover after earthworm invasion was *Corylus cornuta*. This plant is a common mid‐story shrub in sugar maple––basswood forest stands in the Chippewa National Forest, and can form dense, impenetrable thickets in young forests. The *Corylus* genus is known to form symbioses with ectomycorrhizal fungi, and therefore may experience a competitive disadvantage when earthworms become established.

Two other understory woody species that had lower mean cover values in sites that had been wormed for over two decades compared to unwormed/lightly wormed sites were *Acer rubrum* and *Acer spicatum*. Previous studies found that earthworm invasion can have negative effects on *Acer saccharum* colonization (Cassin & Kotanen, 2016; Lawrence et al., 2003), although mean percent cover of understory *Acer saccharum* was greater in long‐term wormed relevés compared to unwormed/lightly wormed relevés in this study. Both *Acer saccharum* and *Acer rubrum* are known to form symbioses with arbuscular mycorrhizae (Brundrett and Kendrick 1988; Wiseman & Wells, 2005). Overall, the *Acer* genus had slightly greater mean cover in short‐term wormed relevés than unwormed/lightly wormed relevés, but in long‐term wormed relevés the *Acer* genus had mean understory cover values less than unwormed/lightly wormed relevés, indicating members of the *Acer* genus are “losers” as earthworms become established over multiple decades.

Mean total cover of the Araliaceae family was lowest in relevés that had been wormed for over two decades. *Aralia nudicaulis* experienced a 45.9% decrease in mean cover in long‐term worm relevés (2.0% cover) when compared to unwormed/lightly wormed releves (3.7%). Both *Aralia nudicaulis* and *Aralia racemosa* are obligately mycorrhizal (Lawrence et al., 2003). In a mesocosm experiment by Hale et al. (2008), *Aralia racemosa* did not experience significantly higher mortality when exposed to earthworms over a period of 13–18 weeks, perhaps echoing our results that decreases in *Aralia racemosa* cover associated with invasive earthworms must be observed over a longer time frame.

The effects of invasive earthworms on mean cover of understory vegetation varied by Küchler life form (woody deciduous, woody evergreen, graminoids, and forbs). Mean woody deciduous cover was lowest in unwormed/lightly wormed sites, followed by long‐term wormed sites. Mean woody deciduous cover was highest in short‐term wormed sites. This phenomenon may be a result of a flush of readily available nutrients available for plant uptake immediately after earthworm invasion, followed by a longer‐term decrease in nutrients as these nutrients become prone to leaching and outwash (Resner et al. 2015). Woody evergreen species had relatively low dominance in all categories, likely because relevés selected for resampling were classified as sugar‐maple basswood forests. Mean cover of forbs was intermediate at unwormed/lightly wormed sites, greatest at short‐term wormed sites, and reached its lowest value at long‐term wormed sites. Forbs may experience an initial flush of growth following a recent earthworm invasion, followed by a longer‐term decrease in total cover (Hale et al., 2008). Like the growth observed in short‐term wormed understory woody deciduous vegetation, the initial pulse of growth observed in short‐term wormed forbs could be due to a buildup of readily available nutrients resulting from mineralization of the O‐horizon (Dobson et al., 2017). The two plant families with the largest total mean negative change in cover from unwormed/lightly wormed to long‐term wormed relevés were both exclusively represented by forbs in our dataset: Asteraceae (43% decrease in mean cover) and Violaceae (48% decrease in mean cover).

Mean total cover of graminoids experienced the greatest degree of change as earthworms became established over time. The greater mean cover values of graminoids that we observed after short‐term and long‐term earthworm invasion mirrors that reported by Craven et al. (2017), who found that total cover of graminoids increased with increasing earthworm biomass. Positive changes in mean understory cover values for the Poaceae family were less pronounced than Cyperaceae (+32% mean cover, a 440% increase), but still showed a net positive change in total mean cover (+1% mean cover, a 28% increase) in unwormed/lightly wormed relevés compared to relevés that had been wormed for over two decades. Graminoid species that possess the following traits may experience a competitive advantage after earthworms become established: tolerance of drought and root herbivory, the presence of bud banks, and non‐obligate mycorrhizal associations (Craven et al., 2017).

### 4.2 Diversity

Similar to findings by Hale et al. (2006), Holdsworth et al. (2007), and Hopfensperger et al. (2011), our study found that understory plant diversity was lowest in sites that had experienced earthworm invasion. Across all diversity metrics measured (Shannon–Wiener, inverse Simpson's, Pielou's evenness, and species richness), long‐term wormed relevés had lower understory plant species diversity than unwormed/lightly wormed and short‐term wormed relevés. Unwormed/lightly wormed relevés exhibited the highest average diversity (Shannon–Wiener, inverse Simpson's, Pielou's evenness, and species richness) at the 10 × 10‐m scale. Species evenness was lowest in sites that had been wormed for over two decades, likely reflecting a greater dominance of *Carex pensylvanica* monocultures.

### 4.3 Species richness and scale

We were not able to find published information about the effects of invasive earthworms on understory plant species richness at multiple scales. In our study, we found that at the smallest scale (1 m^2^) unwormed/lightly wormed species‐area plots had the greatest mean species richness. At this scale, short‐term and long‐term wormed sites had similar numbers of mean species richness. At intermediate scales, unwormed/lightly wormed species‐area plots had the highest mean species richness, followed by short‐term wormed sites, and long‐term wormed sites had the lowest mean species richness. At the largest scale, unwormed/lightly wormed and short‐term sites had similar mean species richness, although unwormed/lightly wormed mean species richness was highest. The lower mean species richness in long‐term wormed sites became more pronounced at the largest scale (1024 m^2^). Our results indicate that sites that have been wormed for multiple decades have, on average, lower species richness at both small (1 m^2^) and large (1024 m^2^) scales. The full extent of reduced species richness associated with earthworm invasion may not be apparent until earthworms have been established for multiple decades.

### 4.4 Plant community composition

Sites that were classified as unwormed/lightly wormed (within‐group) had the lowest dissimilarity, indicating these sites were the most compositionally similar. The second least compositionally dissimilar categories were unwormed/lightly wormed and short‐term wormed sites (between‐group), indicating that these sites also shared a large degree of compositional similarity. Sites that had been wormed for the longest period had the greatest rates of dissimilarity between short‐term wormed (between‐group) and unwormed/lightly wormed (between‐group) relevés. This may indicate that the greatest compositional changes in plant communities that have been invaded by European earthworms occur after two decades or more of invasion. If greater within‐group compositional dissimilarity indicates a community currently in transition, then it appears that both short‐term wormed and long‐term wormed sites are currently in a state of transition. Compositional dissimilarity was greatest at sites that had been wormed for over two decades, and sites that had been wormed for at least two decades did not appear to have reached a compositionally similar end‐state “wormed” community type.

Anecic earthworms feed on both leaf litter and mineral soil and create extensive vertical burrows in the soil. The general sequence of earthworm invasion in the northern Midwest appears to follow the order of *Dendrobaena (epigeic)* > *Lumbricus* juveniles (anecic/epi‐endogeic) > *Aporrectodea* (endogeic) > *Lumbricus terrestris (anecic)* (Holdsworth et al., 2007). Epigeic earthworms invade first, typically followed by anecic, endogeic, and epi‐endogeic earthworm species, subsequently followed by a high biomass of mature anecic earthworms (Loss et al., 2013). Therefore, sites with anecic earthworms present (located in the bottom right corner of the ordination diagram) are more characteristic of late‐stage earthworm invasion.

When analyzing plant community composition in the Chippewa National Forest, it is important to note that invasive earthworms are one of the multiple factors affecting plant community composition. Other factors likely affecting understory plant community composition in the Chippewa National Forest include forest succession and white‐tailed deer (*Odocoileus virginianus*) overabundance. We do not have reason to believe that forest succession (represented by time since the last timber harvest) affected one worm category more than another. None of the forest stands included in this study had been harvested for three decades or more when resampled in summer 2017. We saw no significant differences in canopy cover values among the worming categories. Therefore, our results depict a typical hardwood forest understory plant community at various stages of earthworm infestation that has not recently been harvested.

Our study focused on the effects of one group of organisms (earthworms) on understory plant community composition. Studies have shown that plants respond to multiple co‐occurring stressors, such as invasive earthworms and deer herbivory, simultaneously (Dávalos et al., 2015; Dobson & Blossey, 2015; Fisichelli et al., 2013). Thus, our observed plant community profiles are likely a reflection of a combination of biotic and abiotic stressors, including deer herbivory. Specific data on deer densities at fine scales within the Chippewa National Forest at the time of sampling were not available. Overall, the Leech Lake Reservation deer permit area within the Chippewa National Forest had roughly 15 pre‐fawn deer per square mile in 2017 (Norton & Giudice, 2017). Within the Chippewa National Forest, it is likely that there are localized patches of higher and lower deer densities, considering variations in private, tribal, and federal deer management policies among regions of the Chippewa National Forest. However, we do not have reason to suspect that deer density was higher or lower among the three earthworm categories.

Unwormed hardwood forest stands are becoming increasingly uncommon in the Chippewa National Forest. Despite a concerted effort to locate unwormed permanent vegetation relevés during the summer of 2017, only six relevés were found that remained unwormed or had only leaf‐litter dwelling earthworms present in 1/3 samples. It is essential that baseline data on plant community composition of unwormed sites is collected before these areas disappear, especially given their apparent diversity when compared to sites invaded by earthworms. In the Chippewa National Forest, unwormed sites may persist in isolated areas that function as habitat islands, such as forest stands surrounded by peatlands. These few remaining unwormed areas should be targeted for baseline studies.

If results of this study are indicative of future trends as earthworms become established, it can be expected that understory diversity will decrease as hardwood forest stands become wormed over time. There may be an initial flush of understory diversity immediately post earthworm invasion as nutrients become readily available, however this will likely be followed by a long‐term decrease in understory vegetation diversity. Native understory plant species that form symbioses with mycorrhizal fungi or plants that rely on a thick organic horizon may face a competitive disadvantage where earthworms are present. Rare native plant species which form obligate symbioses with mycorrhizal fungi and are reliant on a thick duff layer, such as *Botrychium* (Gundale, 2002) and *Sceptridium* genera, may experience habitat loss leading to the eventual extirpation of one or more of these rare plants. Plants that may benefit from earthworm invasion include non‐mycorrhizal species and nonnative plants which have coevolved with earthworms. At the time of sampling, shade‐tolerant invasive species such as garlic mustard (*Alliaria petiolata*) and common buckthorn (*Rhamnus cathartica*) were not yet pervasive in our study areas, or in the majority of Mesic hardwood forest stands of the Chippewa National Forest. Further research is needed on the long‐term effects of invasive earthworms on invasive plant species, as these invasive plant species continue to become more prevalent in the forest understory. By understanding the effects of invasive earthworms on understory plant composition over multiple decades, the biological consequences of earthworm invasion can be more effectively predicted and managed as invasive earthworms continue to spread across the Great Lakes region.

## CONFLICT OF INTEREST

The authors declare that they have no conflict of interest.

## CODE AVAILABILITY

Raw data and code are available online at github.com/jinnyalexander/Earthworm_plant_data

## Data Availability

The data that support the findings of this study are openly available in GitHub at https://github.com/jinnyalexander/Earthworm_plant_data.

## Appendix A

### A.1

Percent cover of understory plant species and Küchler life form for unwormed/lightly wormed, short‐term wormed, and long‐term wormed relevés. Data were collected from 41 10 × 10‐m relevés during summer 2017 in the Chippewa National Forest. Sample size (*n*) represents the number of relevés that each plant species was observed. Plant species that occurred in less than six relevés were omitted from the table. An asterisk indicates significant differences at the *p* = 0.05 level. Significance was calculated using the Kruskal–Wallis H test (df = 2)

**Table:**

Mean cover of understory plant species among worm categories				
Species	*n*	Unwormed/lightly wormed	Short‐term Wormed	Long‐term Wormed
Woody deciduous	41	45.0	47.2	46.3
*Acer rubrum* (p* = 0.022*)*	20	3.0	1.1	1.0
*Acer saccharum*	41	10.8	13	12.3
*Acer spicatum*	24	2.7	2.4	1.4
*Amelanchier sp*	15	0.2	0.9	1.5
*Betula papyrifera*	9	0.8	0.2	0.7
*Cornus alternifolia*	30	2.5	2.3	2.3
*Corylus cornuta*	31	4.4	3.1	1.9
*Diervilla lonicera*	6	0.0	0.4	0.5
*Dirca palustris*	28	1.2	2.2	3.1
*Fraxinus pennsylvanica*	22	1.7	1.0	2.0
*Lonicera canadensis*	23	1.5	1.6	2.0
*Lonicera hirsuta*	7	0.5	0.6	0.2
*Ostrya virginiana*	35	2.3	4.6	3.9
*Populus tremuloides*	14	0.5	1.3	2.0
*Prunus virginiana*	27	2.5	2.1	2.6
*Quercus macrocarpa*	22	1.7	1.6	1.1
*Quercus rubra*	23	2.2	1.2	1.8
*Ribes cynosbati*	22	1.5	1.6	1.2
*Ribes triste*	8	0.7	0.6	0.1
*Rubus pubescens*	11	1.5	0.6	1.2
*Tilia americana*	28	2.8	2.4	1.8
*Ulmus americana*	7	0.2	0.5	0.2
*Viburnum rafinesquianum*	12	0.5	1.1	0.8
**Woody evergreen*** (*p* = 0.042)	**15**	**1.5**	**1.0**	**0.1**
*Abies balsamea*	14	0.8	0.9	0.1
**Graminoids*** (*p* = 0.027)	**41**	**11.7**	**23.5**	**45.0**
*Brachyelytrum aristosum*	11	1.0	1.0	0.2
*Carex deweyana*	6	0.5	0.3	0.2
*Carex pedunculata*	32	1.8	3.7	2.9
*Carex pensylvanica* (p* = *0.036)*	41	5.0	13.4	35.8
*Oryzopsis asperifolia*	35	2.8	3.1	4.4
**Forbs**	**41**	**63**	**75.3**	**56.1**
*Actaea rubra*	10	0.5	0.9	0.8
*Amphicarpaea bracteata*	7	0.2	0.8	0.4
*Anemone americana*	19	2.2	1.5	1.8
*Anemone quinquefolia*	33	4.0	3.5	3.1
*Aralia nudicaulis*	33	3.7	3.0	2.0
*Aralia racemosa*	16	0.3	1.3	0.6
*Arisaema triphyllum*	15	0.0	1.1	1.7
*Asarum canadense*	12	0.5	1.8	0.8
*Athyrium filix‐femina*	25	2.5	4.9	1.5
*Botrypus virginianus*	22	1.5	2.0	0.8
*Caulophyllum thalictroides*	7	0.2	0.6	0.2
*Clintonia borealis*	22	2.2	1.9	1.2
*Dendrolycopodium dendroideum*	11	0.5	1.0	0.6
*Dryopteris carthusiana*	10	0.5	0.4	0.8
*Equisetum scirpoides*	6	0.5	0.3	0.9
*Eurybia macrophylla*	32	3.3	3.4	2.0
*Fragaria virginiana*	11	1.3	1.2	0.5
*Galium triflorum*	18	1.5	1.1	1.2
*Lathyrus ochroleucus*	17	1.8	1.3	1.2
*Lysimachia borealis*	27	2.3	2.8	1.6
*Maianthemum canadense*	38	2.8	4.3	4.8
*Maianthemum racemosum*	16	0.7	1.1	1.2
*Osmorhiza claytonii*	28	3.0	2.3	2.1
*Osmunda claytoniana*	10	0.5	1.4	0.1
*Polygonatum pubescens*	31	2.7	3.3	1.5
*Pteridium aquilinum*	12	1.0	1.1	0.9
*Sanicula marilandica*	13	1.0	1.1	0.7
*Solidago flexicaulis*	10	0.7	0.6	0.8
*Streptopus lanceolatus*	33	3.3	3.5	3.4
*Thalictrum dioicum*	18	1.5	1.3	1.2
*Trillium cernuum*	6	0.2	0.4	0.5
*Uvularia grandiflora*	31	3.2	5.8	4.0
*Uvularia sessilifolia*	24	1.8	3.0	1.4
*Viola pubescens/canadensis*	28	2.8	2.1	1.7
*Viola sp/blanda/renifolia/selkirkii/sororia*	11	1.0	1.0	0.1
